# An efficient protocol for perennial ryegrass mesophyll protoplast isolation and transformation, and its application on interaction study between LpNOL and LpNYC1

**DOI:** 10.1186/s13007-017-0196-0

**Published:** 2017-06-05

**Authors:** Guohui Yu, Qiang Cheng, Zheni Xie, Bin Xu, Bingru Huang, Bingyu Zhao

**Affiliations:** 1 College of Agro-grassland Science, Nanjing Agricultural University, Nanjing, 210095 People’s Republic of China; 2 Jiangsu Key Laboratory for Poplar Germplasm Enhancement and Variety Improvement, Nanjing Forestry University, Nanjing, 210037 People’s Republic of China; 3 Department of Plant Biology and Pathology, Rutgers, the State University of New Jersey, New Brunswick, NJ 08901 USA; 4 Department of Horticulture, Virginia Polytechnic Institute and State University, Blacksburg, VA 24061 USA

**Keywords:** Ryegrass, Lolium, Protoplast, Transient gene expression

## Abstract

**Background:**

Perennial ryegrass (*Lolium perenne* L.) is an important temperate grass used for turf and forage purposes. With the increasing accumulation of genomic and transcriptomic data of perennial ryegrass, an efficient protoplast and transient gene expression protocol is highly desirable for in vivo gene functional studies in its homologous system.

**Results:**

In this report, a highly efficient protoplast isolation (5.6 × 10^7^ protoplasts per gram of leaf material) and transient expression (plasmid transformation efficiency at 55.2%) was developed and the detailed protocol presented. Using this protocol, the subcellular locations of two ryegrass proteins were visualized in chloroplasts and nuclei, respectively, and protein–protein interaction between two chlorophyll catabolic enzymes (LpNOL and LpNYC1) was recorded in its homologous system for the first time.

**Conclusion:**

This efficient protoplast isolation and transformation protocol is sufficient for studies on protein subcellular localization and protein–protein interaction, and shall be suitable for many other molecular biology applications where the mesophyll protoplast system is desirable in perennial ryegrass.

## Background

Perennial ryegrass (*Lolium perenne* L.) is the most widely distributed and cultivated turf and forage grass in temperate zones. World-wide consorted programs are working on the molecular genetics of this grass species. The draft genome of this perennial ryegrass was recently published [1] and the assembly of the genome dataset is undergoing. The accumulation of genomic and transcriptomic datasets provided unprecedented opportunity to conduct functional genomic studies in perennial ryegrass. Stable genetic transformation systems have been established in perennial ryegrass, yet the whole transformation takes months to obtain rooted transgenic plants [2, 3].

The plant protoplast system provides a complementary or, sometimes, an alternative way to the stable genetic transformation system for gene functional analysis in many cases, such as protein subcellular localization, in vivo protein–protein and protein-DNA interactions, protein trafficking and signal transduction, etc. Currently, mesophyll protoplast-based transient expression assays are routinely used in biological studies in Arabidopsis (*Arabidopsis thaliana*) [4, 5], maize (*Zea mays*) [4], tabacco (*Nicotiana tabacum*) [6], rice (*Oryza sativa*) [7, 8], *Populus* [9, 10], and cucumber (*Cucumis sativus*) [11]. Successful ryegrass protoplast isolation from mesophyll cells has been reported previously and was used in the study on chloroplast photosynthetic and photorespiratory carbon metabolism [12]. However, its efficiency remains low and not sufficient engouth for protoplast transformation studies according to our preliminary experimental results following the previously published one (data not shown). An efficient transient expression system based on ryegrass mesophyll protoplast transformation was not reported yet.

In this study, a highly repeatable and efficient protocol for mesophyll protoplast isolation and gene transient expression was developed using ryegrass leaves as starting materials. This protocol provides a facile tool for protein subcellular localization and bimolecular fluorescence complementation (BIFC) assays as shown in this study as well as the other in vivo molecular studies where this system is applicable.

## Methods

### Plant material and growth conditions

Perennial ryegrass (cv. Buena vista) was grown in vermiculite: perlite: peat moss (1:3:9) in a growth chamber with temperature set at 25/20 °C (day/night), photosynthetically active radiation (PAR) of 750 µmol photons m^−2^ s^−1^ and 14 h of light per day. The potted plants were watered at about three-day intervals and fertilized with ½ MS minerals (Murashige and Skoog 1962) once a week. It is CRITICAL to water and fertilizer ryegrass plants regularly to obtain fine starting plant material.

### Reagents and solutions

Recipes for the enzyme solution, Modified W5 solution, MMg solution, PEG-Ca^2+^ solution were listed in Table 1. Cellulase ‘Onozuka’ R-10 (Cat. No. BS197A) and macerozyme R-10 (Cat. No. YM-10-1 g) were purchased from Yakult Pharmaceutical Ind. Co., Ltd., Japan. Noting that the enzyme solution was thermally pretreated at 55 °C for 10 min to inactivate nonspecific enzymes before the addition of BSA, CaCl_2_ and KCl solutions.

**Table 1:** Solution recipes for protoplast isolation and transformation

Solution name	Solution composition	Storage	Usage
Enzyme solution (resuspension solution)	10 mM MES, 1.5% (wt/vol) cellulase R10, 0.75% (wt/vol) macerozyme R10, and 20 mM KCl. 10 mM CaCl2, 0.1% BSA, 1–5 mM β-mercaptoe- thanol (optional) and mannitol (0.6 M), pH 5.7	Room temp. (freshly prepared)	Leaf strips lysis
W5 solution	2 mM MES, 154 mM NaCl, 125 mM CaCl2 and 5 mM KCl, pH 5.7	4 °C	Release and wash protoplasts
MMg solution	4 mM MES, 0.4 M mannitol and 15 mM MgCl2, pH 5.7	4 °C	Resuspend protoplast pellet
PEG-Ca^2+^ solution (resuspension solution)	20% (wt/vol) PEG4000, 100 mM CaCl_2_ and mannitol (0.3 M)	Room temp. (freshly prepared)	Transform plasmids (10ug is used in this study) into protoplasts

PEG-4000 (Cat. No. 25322-68-3), D-Mannitol (Cat. No. DH190-2), and Bovine Serum Albumin (BSA) (Cat. No. 9048-46-8) were purchased from Bei Jing Ding Guo Chang Sheng Biotech Co., Ltd., China; MES (Cat. No. E169) was from Ameresco LLC, USA; CaCl_2_·2H_2_O (Cat. No. 10035-04-8) was from Xi long Chemical Co., Ltd., China; and KCl (Cat. No. 7447-40-7), NaCl (Cat. No. 7647-14-5), and MgCl_2_·6H_2_O (Cat. No. 7791-18-6) were from Sinopharm Chemical Reagent Co., Ltd., China.

### Protoplast isolation

Fully expanded leaves (about 10–12 days after leaf emergence) were collected from healthy ryegrass plants and only the middle sectioned leaves were used as the starting material (the tips and bases of leaf blades were removed). A total of 0.4–0.6 g of leave sections were cut into 0.5 mm strips transversely with sharp razors wetted with the enzyme solution, and the leaf strips, once cut off, were immediately emerged into the 6 ml enzyme solution (Table 1). The leaf strips were then vacuumed (0.1 MPa) for 1 h under dark at room temperature. The enzymatic digestion was carried out in a thermal-stable shaker at 30 rpm, 28 °C in dark for about 6 h to the extent that the leaf strips were readily dissembled upon gentle touches with a pipette tip.

The suspended protoplasts were firstly filtered through a layer of cheese clothes and then through a nylon mesh (75 μm), and the residue was washed twice with 10 ml pre-chilled modified W5 solution (Table 1). The filtrate was centrifuged at 100×*g* for 2 min with low acceleration and deceleration speed (BECKMAN, Model Allegra 64R, Hamburg, Germany). The pelleted protoplasts were re-suspended in 10 ml pre-chilled modified W5 solution, and set still for about one hr on ice (or overnight at 4 °C) to allow protoplast to sediment. The precipitated protoplasts were re-suspended in 3 ml MMg solution (Table 1). According to the counted number of viable protoplasts, the suspension was centrifuged down at 100×*g* for 2 min and re-suspended in the MMg solution to a final concentration of ~5 × 10^5^ cells ml^−1^ (depending on experimental purposes, the concentration can be readily achieved up to 7.3 × 10^6^ cells ml^−1^).

Noting that the pipette tips used in transferring protoplasts should be cut with scissors to minimize mechanical damage.

### Vector construction and plasmid preparation

One chloroplast-localized protein LpPPH [13] and one unnamed NAC transcription factor LpNACx (unpublished result) were used for the subcellular localization test. In brief, the *LpPPH* and *LpNACx* genes were inserted into the p2GWF7.0 vector [14] in fusion with a GFP tag at the C-terminal. For bimolecular fluorescence complementation (BiFC) test, putative *LpNOL* and *LpNYC1* genes were amplified from perennial ryegrass (unpublished), and cloned into a pair of split citrine vectors (pN-citrine-GW and pC-citrine-GW) to generate fusion proteins with citrine N-terminal or with citrine C-terminal tags. The pair of BiFC vectors were constructed in Dr. Bingyu Zhao’s lab at Virginia Tech: the pN-citrine-GW vector was generated using the backbone of p2GW7.0 [15] with an insertion of citrine-N-terminal with a T7 tag in the C terminal of a GATEWAY ccdB cassette(B) which would result in a translational fusion of target protein and citirine-T7 after LR clonase recombination (Invitrogen, Carlsbad, CA); likewise, the pC-citrine-GW vector was constructed with the insertion of citrine-C-terminal with HA tag. The primers used in gene cloning were presented in Table 2. The constructed vectors were electro-transformed into *E. coli* T1 cells (Invitrogen) and the plasmids were extracted using a commercial midi-prep kit (TIANpure plasmid kit II, TianGen Co., Beijing, China) to yield quality DNAs (>300 ng ul^−1^).

**Table 2:** Primers used in gene cloning and vector construction

Primer name	Sequence (5′–3′)
*LpPPH* forward	ATCAGGAATTCATGGAAGTGGTTTCCTCCAG
*LpPPH* reverse	AACCGTCGACAGACACTACCCGTATGTTGGAG
*LpNOL* forward	ATTAGGGATCCATGGCCACCGTCGCCGCC
*LpNOL* reverse	TATTGAAGCTTATCCTCAGTAACATACTTGTTCCTACG
*LpNYC1* forward	AACCAGGATCCATGGCCGCCGCGGTCGTC
*LpNYC1* reverse	AATGAAAGCTTTGTGCCAGGGAAAGGTCCAC
*NAC* forward	TTCATGGATCCATGGCCACTGCCGCTTCC
*NAC* reverse	TATAGAAGCTTCTGCGGCGGCTGGCCGGC
35S forward	AACGGATCCGGTACCCATGGAGTCAAAGATTCAAATAG
35S reverse	AACAAGCTTAGTCCCCCGTGTTCTCT

### PEG-mediated protoplast transformation

The transient gene expression system using ryegrass protoplasts was modified from a protocol reported by Yoo et al. [5]. The optimal amount of plasmid (from 1 to 10 µg) and concentration of mannitol (from 0.1 to 0.3 M) were tested. In brief, plasmids were mixed with 200 µl protoplast stock at RT, adding equal volume of PEG-Ca^2+^ medium (Table 1), gently mixed and setting still for 5 min at RT for plasmid transformation. Then, three ml W5 solution was added slowly and gently mixed with the protoplast suspension, followed by a centrifugation at 100×*g* for 2 min and the pellet was re-suspended gently in 0.5 ml of W5 solution. Finally, the protoplast suspension was transferred into 1.5 ml tubes that were pre-coated with 1% BSA to avoid protoplast attachment to the tube surface, and incubated for 10–24 h in dark at RT.

### Microscopy

The density and viability of isolated protoplasts were counted by using the standard hemocytometer and FDA staining assay [16] and observed under a light microscope (OLYMPUS Model BX53, Tokyo, Japan).

Transformed protoplasts were observed with a confocal laser scanning microscope (Zeiss LSM780 Exciter) for GFP, CFP (citrine), and chloroplast auto-fluorescence with the excitation wavelengths and emission filters set at 488 nm/band-pass 505–530 nm for GFP, 458 nm/band-pass 465–530 nm for CFP, and 488 nm/band-pass 650–710 nm for chloroplast auto-fluorescence. Image processing was performed using the Volocity software (Zeiss).

## Results and discussion

### Protoplast isolation from mesophyll cells of perennial ryegrass

**Fig. 1:**
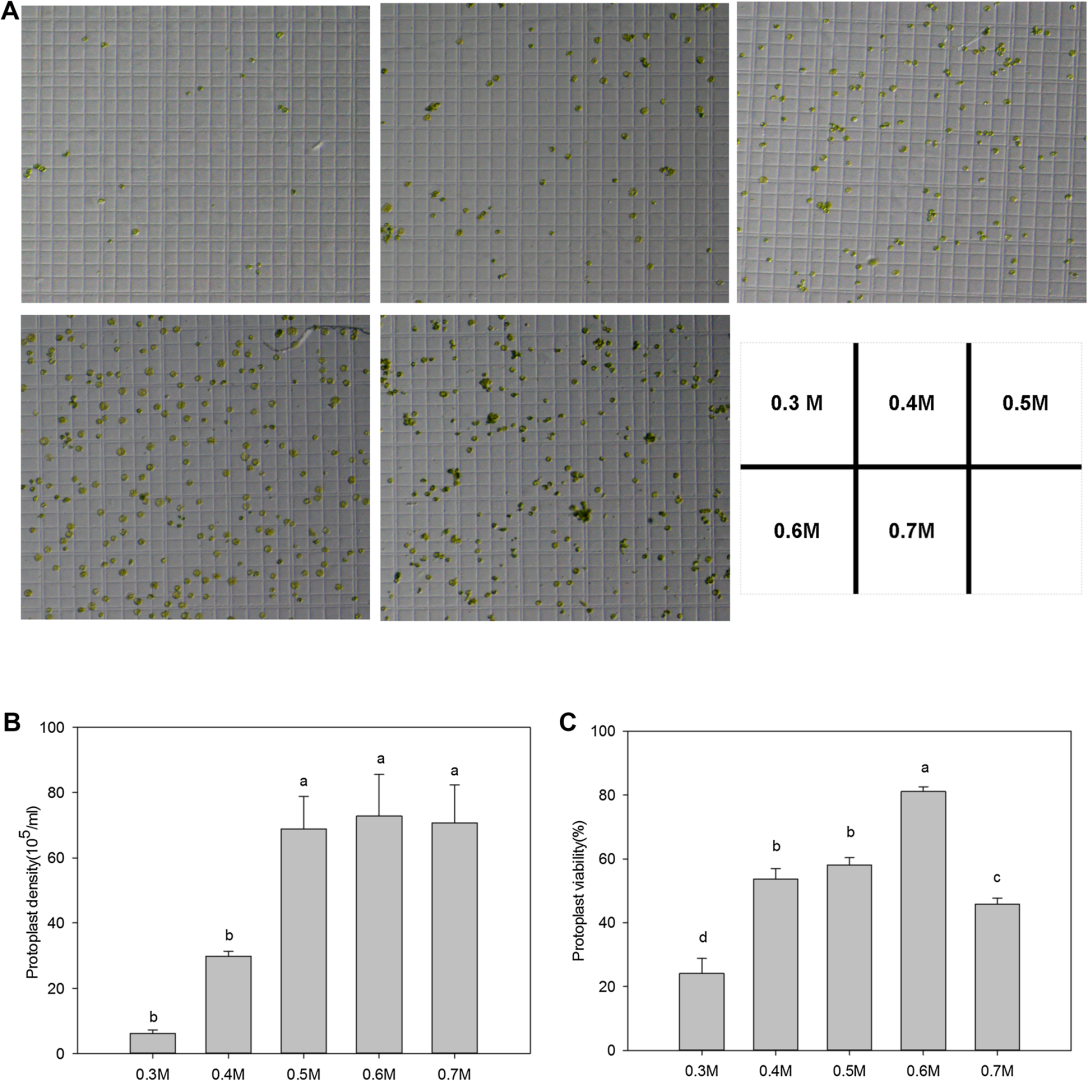
Effects of mannitol concentration on ryegrass protoplast isolation. **A** Isolated protoplast on a hemocytometer; **B** effect of mannitol concentration on protoplast density; **C** effect of mannitol concentration on protoplast viability counted using the FDA staining assay. Mannitol concentration was set at 0.3, 0.4, 0.5, 0.6, or 0.7 M, respectively. The number of intact and viable protoplasts was counted visually for round and intact protoplasts under a light microscope (OLYMPUS Model BX53, Tokyo, Japan). At least 30 protoplasts were counted in one scope, and the means were from ≥3 scopes. *Different letters* represent statistically significant difference at *p* = 0.05, and *bars above columns* represent standard errors

Selecting the proper starting leaf material was critical for the whole protocol. Using unselective green leaves (the 1st to the 3rd leaves from the top) for the protoplast isolation and transient gene expression assay (e.g. for the detection of subcellular localization) yielded inconsistent results. While consistent result was only achieved with the newly and fully expanded leaves (the 2nd leaf from the top which is ~10–12 days after leaf emergence) from healthy plants. The optimum concentrations of cellulose R-10 and macerozyme R-10 used in this study on ryegrass were the same as those used in rice [8], maize [4] and wheat [17]. The concentration of mannitol in the enzyme solution was critical for integrity of isolated protoplasts [18]. After testing a series of mannitol concentrations in the enzyme solution (1.50% cellulose R-10 and 0.75% macerozyme R-10), we found that mannitol at 0.6 M lead to the highly protoplast isolation efficiency (5.6 × 10^7^ protoplasts per gram; Fig. 1) and protoplast viability (82.8%) after enzyme digestion (Fig. 1). After further removal of broken cell debris using centrifugation, the re-suspended protoplast density can be readily achieved at 7.3 × 10^6^ protoplasts per ml (Fig. 1). Compared to the previous ryegrass protoplast isolation protocol [12], the current protocol used different digestive enzyme mix and mannitol concentration with optimized experimental procedures (see notes and details in “Methods” section), which were all indispensable factors for successful and efficient ryegrass protoplast isolation.

### PEG-mediated protoplast transformation

The isolated protoplasts were subjected to PEG-mediated transformation. After adjusting variable parameters, we achieved up to 55.2% transformation efficiency with 10 µg plasmid DNA (plasmid size ~10 kb) prepared with a regular plasmid midi-prep kit. Higher protoplast transformation efficiency could be achieved using higher amount or purity (e.g. with CsCl purified plasmid DNA) of plasmid DNA [5]. The mannitol concentration in the PEG-Ca^2+^ solution was also critical for the transformation efficiency that the highest efficiency was achieved at 0.3 M mannitol (data not shown).

**Fig. 2:**
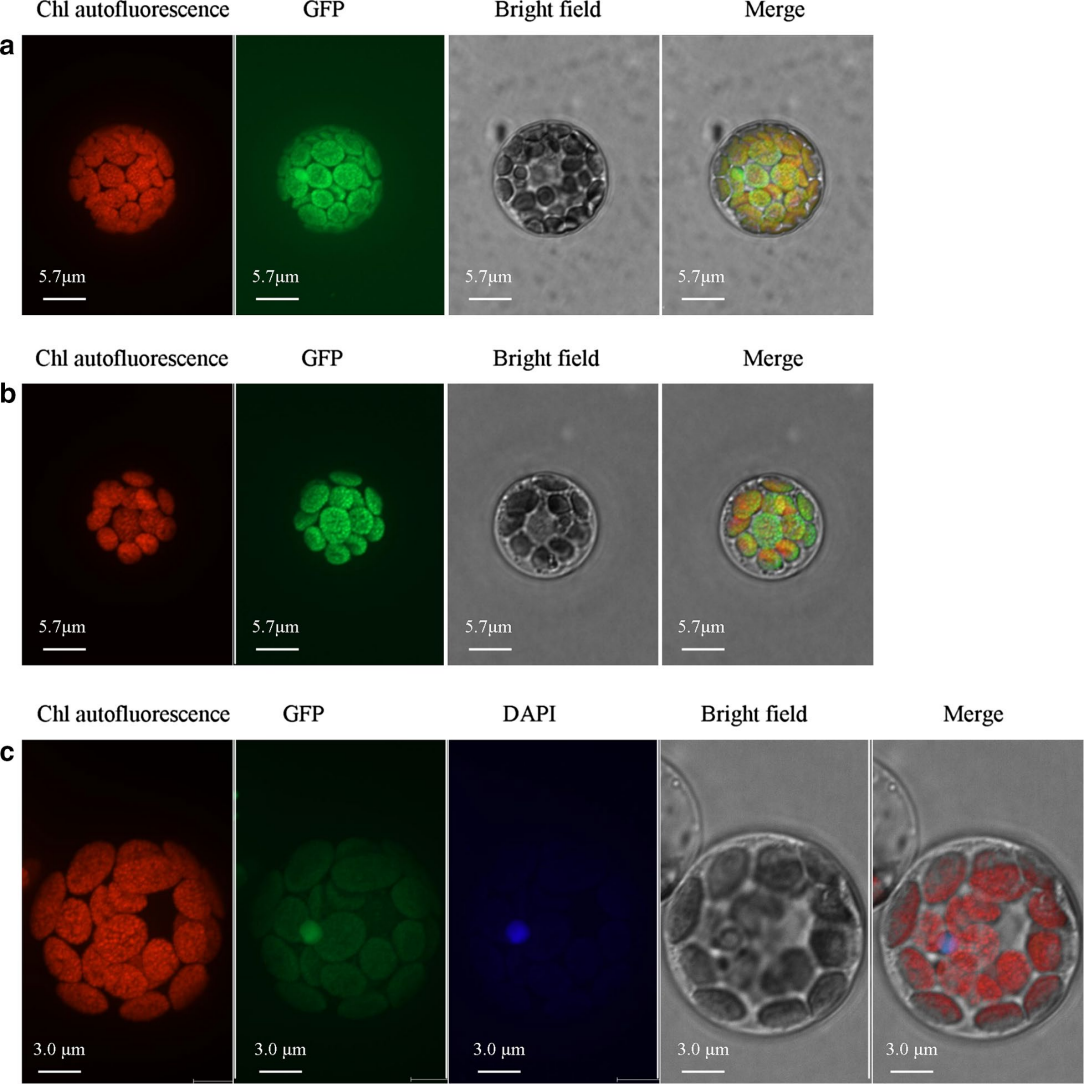
Subcellular localization for different vector in ryegrass protoplasts. **a** 2 × 35 s::*GFP*; **b** 2 × 35 s::*LpPPH*-*GFP*; **c** 2 × 35 s::*LpNAC*-*GFP*

The developed protocol can be efficiently applied to protein subcellular localization and BiFC assays. For examples, LpPPH, a ryegrass chlorophyll catabolic enzyme, was previously shown to localize in chloroplast using Arabidopsis protoplast [13]; using the current protocol, we were able to confirm its subcellular localization in its homologous system (Fig. 2). In another experiment, a putative ryegrass NAC transcription factor was shown localized in the nucleus in ryegrass protoplast (Fig. 2).

### LpNOL and LpNYC1 interact in vivo using BIFC

**Fig. 3:**
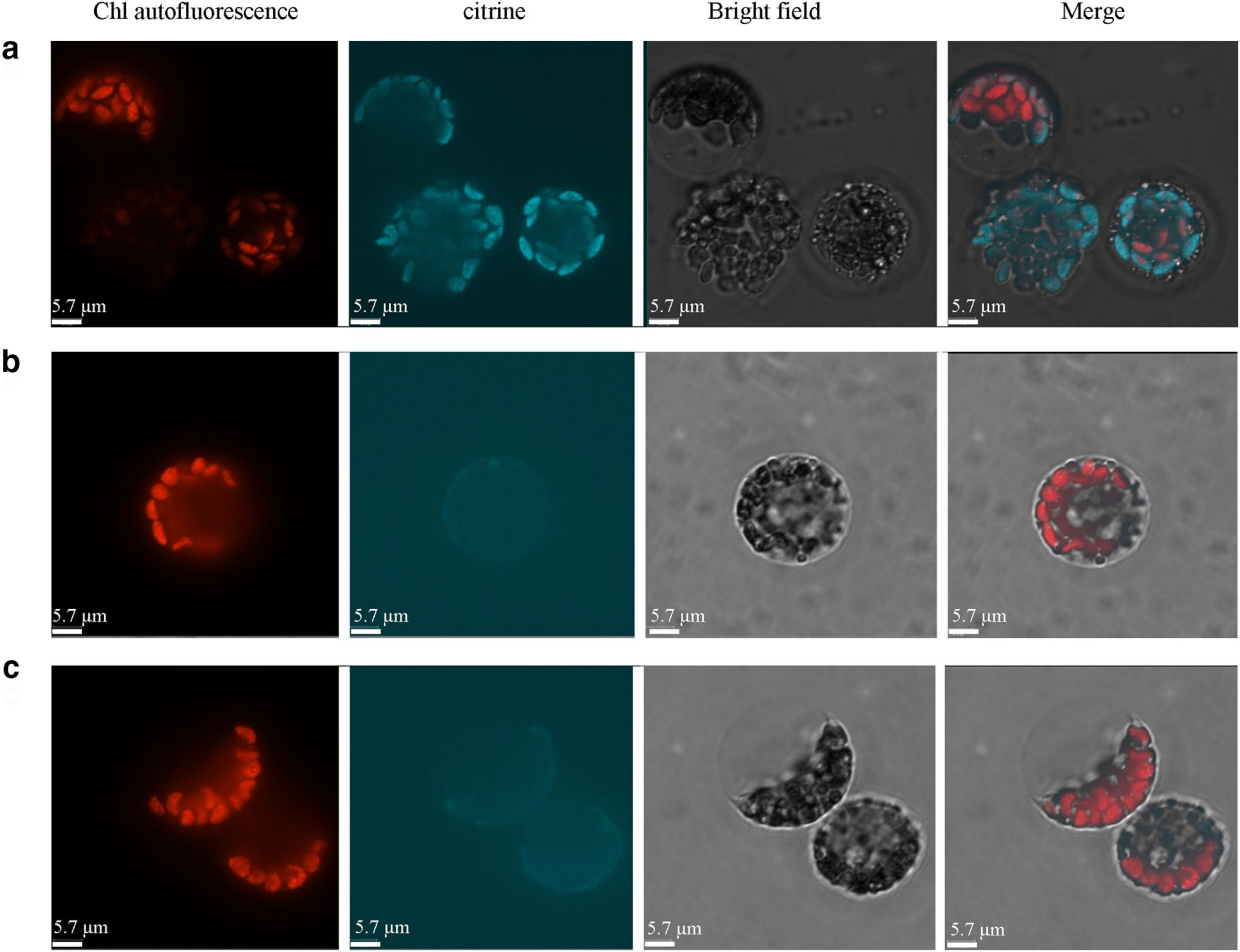
BiFC assay shows the interaction between LpNOL and LpNYC1 in chloroplast. **a** Co-transformed with N-LpNOL + C-LpNYC1; **b** N-LpNOL; **c** N-LpNOL + C-GUS. The genes were in fusion with split citrine (N– or C– terminals). The *bar* represents 5.7 µm

The current protocol is also efficient enough for protein–protein interaction assay (e.g. BIFC). We cloned two putative chlorophyll catabolic enzyme-encoding genes, *LpNOL* & *LpNYC1*, from perennial ryegrass, and fused them with split N- and C- terminal citrine fragments, respectively. Orthologs of these two genes in model plant species Arabidopsis and rice encode key chlorophyll catabolic enzymes (CCEs), which physically interact with each other and cooperatively catalyze the degradation of Chl b [19, 20]. As shown in Fig. 3, the citrine signal was only visualized in the chloroplasts when *LpNOL* & *LpNYC1* were co-transformed, proving that these two proteins interact with each other in vivo in its homologous system.

## Conclusions

**Fig. 4:**
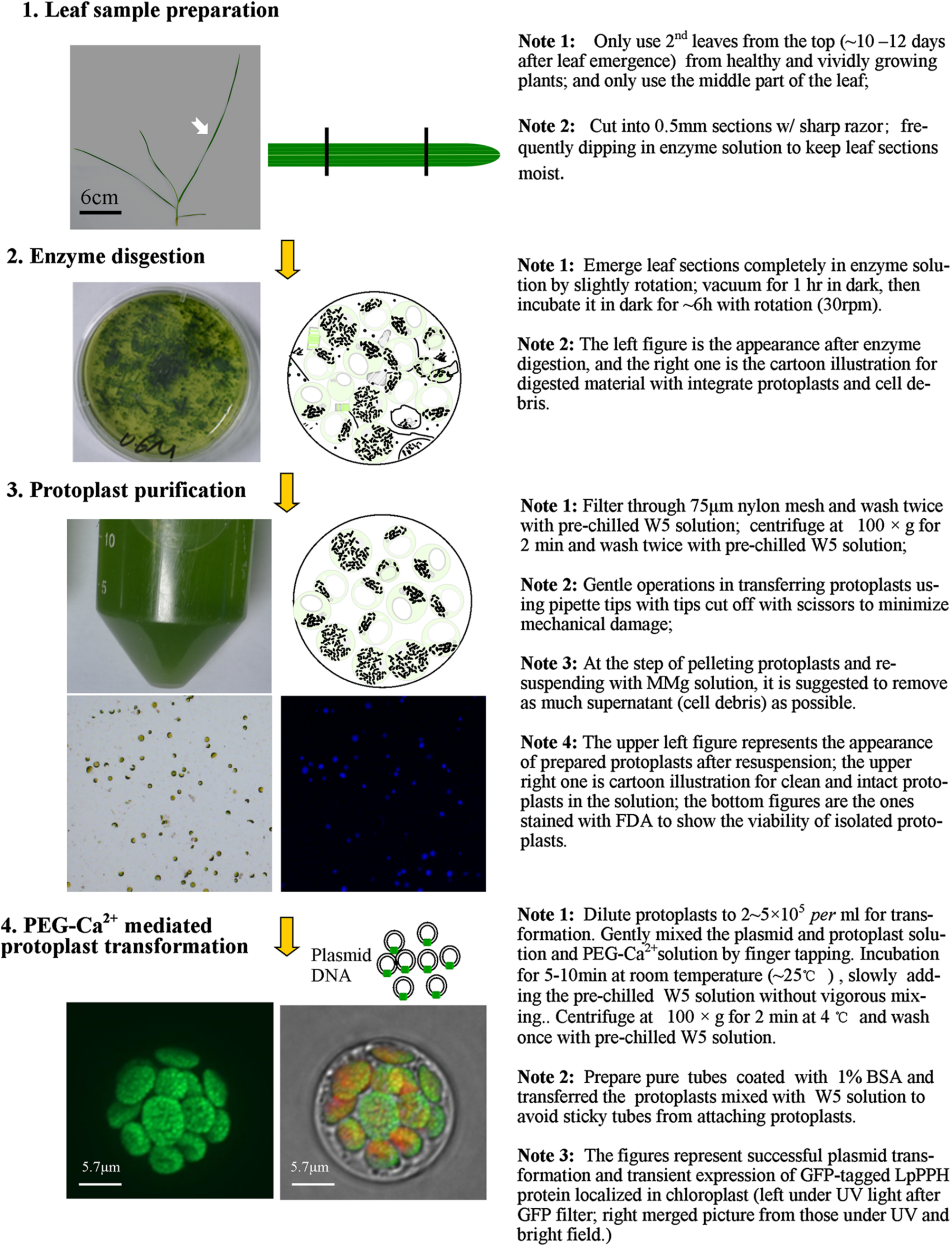
Outline of protoplast isolation and transformation

In sum, a highly efficient mesophyll cell protoplast isolation and transformation protocol was developed for perennial ryegrass, which can be readily used for protein subcellular localization, and protein–protein interaction analysis. The protocol was illustrated in Fig. 4 with critical points pinpointed, and the recipes were shown in Table 1.
